# Supplementary material for: Decision-making flexibility in New Caledonian crows, young children and adult humans in a multi-dimensional tool-use task
Source: PLoS One. 2020 Mar 11;15(3):e0219874. doi: 10.1371/journal.pone.0219874 (PMC7065838; doi:10.1371/journal.pone.0219874)
Supplement: S1 Table — In the tool selection and apparatus functionality condition the total number of trials (in brackets) varied between individuals, depending on when they reached the criterion. P-values calculated from exact binomial tests. Significant p-values highlighted in bolt. MPR = most preferred reward, Sessions 1 to 2 in the tool selection and tool selection quality allocation condition are for comparability with the Goffin’s cockatoos; * p < 0.05, ** p <0.01, *** p < 0.001. (DOCX) [file pone.0219874.s001.docx]

**S1 Table. Number of correct trials for all constellations of the tests for the crows for each individual.** In the tool selection and apparatus functionality condition the total number of trials (in brackets) varied between individuals, depending on when they reached the criterion. P-values calculated from exact binomial tests. Significant p-values highlighted in bolt. MPR = most preferred reward, Sessions 1 to 2 in the tool selection and tool selection quality allocation condition are for comparability with the Goffin’s cockatoos; * p < 0.05, ** p <0.01, *** p < 0.001

| **Condition** | **Apparatus** | **Grouping of tools and reward** | **Individuals** | | | | | |
| --- | --- | --- | --- | --- | --- | --- | --- | --- |
|  |  |  | **Janis** | **David** | **Freddie** | **Elvis** | **Annie** | **Bob** |
| Tool Selection | Stick | Session 1 & 2 | **10*** | **10*** | **11**** | **10*** | **11**** | **10*** |
|  | Stone |  | 5 | 5 | 6 | 9 | 4 | 8 |
|  | Stick | All sessions | **36*** (42)** | **15** (18)** | **16** (18)** | **15** (18)** | **22* (30)** | **15** (18)** |
|  | Stone |  | 25 (42) | 9 (18) | 13 (18) | **15** (18)** | **21* (30)** | 13 (18) |
| Motivation | Stick | Tool | 0 | 0 | 0 | 0 | 0 | 0 |
|  |  | Food | **12***** | **12***** | **12***** | **12***** | **12***** | **12***** |
|  | Stone | Tool | 0 | 0 | 0 | 0 | 2 | 0 |
|  |  | Food | **12***** | **12***** | **12***** | **12***** | **10*** | **12***** |
| Quality Allocation | Stick | MPR Inside | 7 | **12***** | 0 | **12***** | **11**** | **12***** |
|  |  | MPR Outside | **12***** | **11**** | **12***** | **12***** | **12***** | **11**** |
|  | Stone | MPR Inside | 8 | **12***** | 1 | **12***** | **12***** | **12***** |
|  |  | MPR Outside | **12***** | **11**** | **12***** | **12***** | **12***** | **12***** |
| Tool Functionality | Stick | Functional | 6 | **12***** | 5 | **12***** | **10*** | **12***** |
|  |  | Non-functional | **12***** | 0 | 7 | 0 | 2 | 0 |
|  | Stone | Functional | 6 | **12***** | 9 | **12***** | **12***** | **12***** |
|  |  | Non-functional | **10*** | 1 | 9 | 1 | 1 | 1 |
| Tool Selection Quality Allocation | Stick | Session 1 & 2 | 4 | 6 | 4 | **11**** | 4 | 8 |
|  | Stone |  | 6 | **10*** | 9 | **10*** | **12***** | 8 |
|  | Stick | All sessions | 27 | 28 | 22 | **46***** | 21 | **35**** |
|  | Stone |  | **33*** | **40***** | **44***** | **46***** | **44***** | **37***** |
| Apparatus Functionality | Stick | All sessions | **23** (30)** | **40* (60)** | 13 (18) | **12*** (12)** | 16 (36) | **10* (12)** |
|  | Stone |  | **25*** (30)** | **45*** (60)** | **17*** (18)** | **12*** (12)** | **35*** (36)** | **11** (12)** |
| Apparatus Choice | Stick | 2 Sessions | **10*** | **12***** | **11**** | **11**** | **12***** | **12***** |
|  | Stone |  | **11**** | **12***** | **12***** | **12***** | **12***** | **12***** |
