# Supplementary material for: Decision-making flexibility in New Caledonian crows, young children and adult humans in a multi-dimensional tool-use task
Source: PLoS One. 2020 Mar 11;15(3):e0219874. doi: 10.1371/journal.pone.0219874 (PMC7065838; doi:10.1371/journal.pone.0219874)
Supplement: S2 Table — (DOCX) [file pone.0219874.s002.docx]

**S2 Table. Crow subject information**

| **Name** | **Sex** | **Age** |
| --- | --- | --- |
| Janis | female | adult |
| Freddie | male | juvenile |
| Elvis | male | adult |
| David | male | adult |
| Annie | female | juvenile |
| Bob | male | adult |
