# Supplementary material for: Decision-making flexibility in New Caledonian crows, young children and adult humans in a multi-dimensional tool-use task
Source: PLoS One. 2020 Mar 11;15(3):e0219874. doi: 10.1371/journal.pone.0219874 (PMC7065838; doi:10.1371/journal.pone.0219874)
Supplement: S3 Table — N = 6. Significant p-values are highlighted in bold. (DOCX) [file pone.0219874.s003.docx]

**S3 Table. Generalized linear mixed models on factors affecting the number of correct trials in crows**. N = 6. Significant p-values are highlighted in bold.

|  | Estimate | z value | p-value |  |
| --- | --- | --- | --- | --- |
| **(Intercept)** | **3.780** | **5.932** | ***< .001*** | ******* |
| **Apparatus functionality** | **-2.595** | **-4.336** | ***< .001*** | ******* |
| Motivation | 0.413 | 0.449 | *.654* |  |
| **Quality allocation** | **-1.881** | **-3.078** | ***.002*** | ****** |
| **Tool functionality** | **-3.580** | **-6.005** | ***< .001*** | ******* |
| **Tool selection quality allocation** | **-2.882** | **-4.875** | ***< .001*** | ******* |
| **Tool selection** | **-2.753** | **-4.591** | ***< .001*** | ******* |
| Sex | 0.194 | 0.757 | *.449* |  |
| Trial | 0.016 | 0.988 | *.323* |  |
| Age | -0.347 | -1.354 | *.176* |  |
