# Supplementary material for: Decision-making flexibility in New Caledonian crows, young children and adult humans in a multi-dimensional tool-use task
Source: PLoS One. 2020 Mar 11;15(3):e0219874. doi: 10.1371/journal.pone.0219874 (PMC7065838; doi:10.1371/journal.pone.0219874)
Supplement: S4 Table — (DOCX) [file pone.0219874.s004.docx]

**S3-1: Posthoc comparison of conditions of crow data with Tukey correction for multiple comparison**

| **Comparison** | | | **Estimate** | **z value** | **Pr(>\|z\|)** |  |
| --- | --- | --- | --- | --- | --- | --- |
| Apparatus functionality | vs. | Apparatus choice | -2.596 | -4.336 | < 0.001 | *** |
| Motivation | vs. | Apparatus choice | 0.413 | 0.449 | 0.999 |  |
| Quality allocation | vs. | Apparatus choice | -1.881 | -3.078 | 0.026 | * |
| Tool functionality | vs. | Apparatus choice | -3.580 | -6.005 | < 0.001 | *** |
| Tool selection quality allocation | vs. | Apparatus choice | -2.882 | -4.875 | < 0.001 | *** |
| Tool selection | vs. | Apparatus choice | -2.753 | -4.591 | < 0.001 | *** |
| Motivation | vs. | Apparatus functionality | 3.009 | 4.155 | < 0.001 | *** |
| Quality allocation | vs. | Apparatus functionality | 0.715 | 3.179 | 0.019 | * |
| Tool functionality | vs. | Apparatus functionality | -0.984 | -5.444 | < 0.001 | *** |
| Tool selection quality allocation | vs. | Apparatus functionality | -0.287 | -1.751 | 0.530 |  |
| Tool selection | vs. | Apparatus functionality | -0.158 | -0.825 | 0.978 |  |
| Quality allocation | vs. | Motivation | -2.294 | -3.123 | 0.023 | * |
| Tool functionality | vs. | Motivation | -3.993 | -5.530 | < 0.001 | *** |
| Tool selection quality allocation | vs. | Motivation | -3.296 | -4.589 | < 0.001 | *** |
| Tool selection | vs. | Motivation | -3.166 | -4.367 | < 0.001 | *** |
| Tool functionality | vs. | Quality allocation | -1.699 | -7.799 | < 0.001 | *** |
| Tool selection quality allocation | vs. | Quality allocation | -1.001 | -4.901 | < 0.001 | *** |
| Tool selection | vs. | Quality allocation | -0.872 | -3.834 | 0.002 | ** |
| Tool selection quality allocation | vs. | Tool functionality | 0.698 | 4.545 | < 0.001 | *** |
| Tool selection | vs. | Tool functionality | 0.827 | 4.502 | < 0.001 | *** |
| Tool selection | vs. | Tool selection quality allocation | 0.129 | 0.772 | 0.984 |  |
