# Supplementary material for: Decision-making flexibility in New Caledonian crows, young children and adult humans in a multi-dimensional tool-use task
Source: PLoS One. 2020 Mar 11;15(3):e0219874. doi: 10.1371/journal.pone.0219874 (PMC7065838; doi:10.1371/journal.pone.0219874)
Supplement: S5 Table — N = 88. Significant p-values are highlighted in bold. (DOCX) [file pone.0219874.s005.docx]

**S4 Table. Generalized linear mixed models on factors affecting the number of correct trials in children aged 3-5 years, with age in years**. N = 88. Significant p-values are highlighted in bold.

| **Fixed term** | **Estimate** | **z-value** | **p-value** |
| --- | --- | --- | --- |
| Quality allocation | -0.02443 | 1.35745 | *.986* |
| Tool functionality | 0.91738 | 1.36632 | *.502* |
| Tool selection quality allocation | -0.06341 | 1.74130 | *.971* |
| Motivation | -0.34124 | 1.65036 | *.836* |
| **Age** | **0.93910** | **0.31758** | ***.003*** |
| Gender | 0.16391 | 0.13613 | *.229* |
| Trial | 0.09053 | 0.07170 | *.207* |
| Quality allocation:Age | -0.19971 | 0.36531 | *.585* |
| **Tool functionality:Age** | **-0.84813** | **0.34353** | ***.016*** |
| Tool selection quality allocation:Age | -0.25778 | 0.42943 | *.548* |
| **Motivation:Age** | **-0.92291** | **0.37470** | ***.014*** |
