# Supplementary material for: Decision-making flexibility in New Caledonian crows, young children and adult humans in a multi-dimensional tool-use task
Source: PLoS One. 2020 Mar 11;15(3):e0219874. doi: 10.1371/journal.pone.0219874 (PMC7065838; doi:10.1371/journal.pone.0219874)
Supplement: S6 Table — (DOCX) [file pone.0219874.s006.docx]

**S4-1: Posthoc comparison of conditions of children data with Tukey correction for multiple comparison**

| **Comparison** | | | **Estimate** | **z-value** | **Pr(>\|z\|)** |
| --- | --- | --- | --- | --- | --- |
| Quality allocation | vs. | Tool selection | -0.02443 | -0.018 | 1.0 |
| Tool functionality | vs. | Tool selection | 0.91738 | 0.671 | 0.960 |
| Tool selection quality allocation | vs. | Tool selection | -0.06341 | -0.036 | 1.0 |
| Motivation | vs. | Tool selection | -0.34124 | -0.207 | 1.0 |
| Tool functionality | vs. | Quality allocation | 0.94181 | 1.030 | 0.832 |
| Tool selection quality allocation | vs. | Quality allocation | -0.03898 | -0.028 | 1.0 |
| Motivation | vs. | Quality allocation | -0.31681 | -0.256 | 0.999 |
| Tool selection quality allocation | vs. | Tool functionality | -0.98079 | -0.795 | 0.927 |
| Motivation | vs. | Tool functionality | -1.25862 | -1.218 | 0.727 |
| Motivation | vs. | Tool selection quality allocation | -0.27783 | -0.203 | 1.0 |
