# Supplementary material for: Decision-making flexibility in New Caledonian crows, young children and adult humans in a multi-dimensional tool-use task
Source: PLoS One. 2020 Mar 11;15(3):e0219874. doi: 10.1371/journal.pone.0219874 (PMC7065838; doi:10.1371/journal.pone.0219874)
Supplement: S7 Table — Results reflect results of Wilcoxon 1-sample signed ranks tests–chance value = 50%. Significant p-values (<0.05) highlighted in bold. (DOCX) [file pone.0219874.s007.docx]

**S5 Table. Performance across all conditions for the crows with each apparatus separately**. Results reflect results of Wilcoxon 1-sample signed ranks tests – chance value = 50%. Significant p-values (<0.05) highlighted in bold.

| **Apparatus Types Singly** |  | **Stone** | **Stick** |
| --- | --- | --- | --- |
| Tool selection (S1&2) | T^+^ | 8.5 | 21 |
|  | *p* | *.785* | ***.023*** |
| Motivation | T^+^ | 21 | 21 |
|  | *p* | ***.02*** | ***.014*** |
| Quality Allocation | T^+^ | 21 | 15 |
|  | *p* | ***.026*** | ***.042*** |
| Tool functionality | T^+^ | 11 | 8.5 |
|  | *p* | *.332* | *.317* |
| Tool selection quality allocation (S1&2) | T^+^ | 12 | 12 |
|  | *p* | *.116* | *.221* |
| Tool selection quality allocation (S3&4) | T^+^ | 20 | 14.5 |
|  | *p* | ***.046*** | *.4* |
| Tool selection quality allocation  (S5-8) | T^+^ | 21 | 15 |
|  | *p* | ***.027*** | ***.043*** |
| Apparatus functionality | T^+^ | 21 | 10 |
|  | *p* | ***.027*** | *.5* |
| Apparatus choice | T^+^ | N/A | |
|  | *p* |  |  |
