# Supplementary material for: Decision-making flexibility in New Caledonian crows, young children and adult humans in a multi-dimensional tool-use task
Source: PLoS One. 2020 Mar 11;15(3):e0219874. doi: 10.1371/journal.pone.0219874 (PMC7065838; doi:10.1371/journal.pone.0219874)
Supplement: S8 Table — Results reflect Mann Whitney U-tests. Significant p-values highlighted in bold. (DOCX) [file pone.0219874.s008.docx]

**S6 Table. Comparison of performance within conditions between crows and cockatoos.** Results reflect Mann Whitney U-tests. Significant p-values highlighted in bold.

| **Apparatus Types: Combined & Singly** | **Tool selection** | | **Motivation** | | **Quality Allocation** | | **Tool functionality** | | **Tool selection quality allocation** | |
| --- | --- | --- | --- | --- | --- | --- | --- | --- | --- | --- |
|  | U, z | *p* | U, z | *p* | U, z | *p* | U, z | *p* | U, z | *p* |
| Stone | 7,  -2.83 | ***.003*** | 11,  -2.57 | ***.011*** | 19.5,  -1.72 | *.090* | 10.5, -2.52 | ***.010*** | 19.5, -1.72 | *.091* |
| Stick | 32,  -0.641 | *.542* | 6,  -3.02 | ***.002*** | 18,  -1.85 | *.067* | 9,  -2.69 | ***.005*** | 36, -0.27 | *.827* |
| Both | 16.5,  -1.99 | ***.046*** | 8,  -2.8 | ***.004*** | 19,  -1.76 | *.086* | 5,  -3 | ***.001*** | 13, -2.3 | ***.020*** |
