# Supplementary material for: Decision-making flexibility in New Caledonian crows, young children and adult humans in a multi-dimensional tool-use task
Source: PLoS One. 2020 Mar 11;15(3):e0219874. doi: 10.1371/journal.pone.0219874 (PMC7065838; doi:10.1371/journal.pone.0219874)
Supplement: Attachment — Submitted filename: Review for PONE-D-19-18492.docx [file pone.0219874.s011.docx]

Review for

PLOS ONE

Decision-making flexibility in New Caledonian crows, young children and adult humans

in a multi-dimensional tool-use task

This study set out to investigate the ability of human adults, children, and New Caledonian crows to make profitable decisions in a variety of tool-use contexts. In addition, the authors compare their results with the findings from a previous study on cockatoos and orangutans which used a similar metholodogy. Therefore, one of the additions that the current papers brings to the literature is the use of a comparable methodology of a variety of species, including tool-using and non-tool-using ones.

The authors predicted that the crows would show a similar performance as the performance in the orangutans and cockatoos, but had no directed hypothesis about the performance of the human children. The key message that the authors draw from their results is that crows, human adults and children from 4 years of age can make profitable decisions in their studied tool-use context; however, crows show a decreased performance when they had to attend to several cues in parallel and 3-year-old children showed chance performance in almost all conditions. Lastly, the authors found a strong bias for selecting a tool, even if this choice was less efficient, across children of all ages.

The article is appropriately structured and clearly presented. However, I have one small comment on one of the subheadings in the result section (“Performance in New Caledonian crows, Goffin’s cockatoos, orangutans, children and adult humans”) which is formulated a bit too vague (see below). The paper title does reflect the content and attracts attention. All in all, the article fits with the scope of PlosOne.

Please find my detailed comments below.

**Introduction**

The introduction lacks a little bit structure. Several concepts are introduced within the first page (effective decision making, self-control, delay of gratification, tool use, delay of gratification in tool use), which makes it hard to figure out what the primary focus of the current paper is. The overall area of interest is effective decision-making and it is stated that one aspect underlying this ability is self-control. The authors also mention other aspects such as work-effort sensitivity and attention to the functionality of the tools. Even though the latter two concepts are mentioned further below in the paper as well, the introduction suggests that the main focus of the authors is on self-control/delay of gratification. However, as the methods section shows, this is not the case as only some of the studied conditions are explicitly looking at delay of gratification. Others look at the perception of tool functionality and work-effort sensitivity. Therefore, the introduction could be rephrased in stating that several aspects of decision-making in a tool-use context are being investigated. Otherwise, if the focus of the introduction remains heavily on self-control, the choice of that many conditions (some of which do not involve delay of gratification) remains unjustified. This will imply that the authors give some more information on what we already know about work-effort sensitivity and attention to the functionality of tools in the studied species (especially crows and human children).

What I would also find interesting is some statement on how these different concepts of decision-making (self-control, work-effort sensitivity, sensitivity to tool functionality, etc) relate to each other.

What is also lacking at the end of the introduction or at the beginning of the methods section is an account of how the researchers are aiming to measure delay of gratification, work-effort sensitivity, and the sensitivity to the functionality of the tool. That is, here the different conditions/comparisons between conditions can be introduced (in the current paper, the conditions are just described in the methods section, but is remains somewhat implicit what the conditions are designed to measure).

More specific comments I had are:

Lines 58/59: “There is significant improvement by 4 years old and above (14), with some studies showing improvement between ages 3 and 5.” This statement is a bit unclear as it doesn’t become clear how the improvement looks like.
In addition, “There is significant improvement by 4 years “seems to describe almost the same as “improvement between 3 and 5”, and this redundancy is even more pronounced by the fact that the sentence before this one already states that self-control being present in toddlerhood and preschool age. Thus, I suggest to rephrase this sentence to state clearly which improvement the cited papers have shown and how the developmental trajectory looks like. Also note that there is not only evidence for an increase in self-control between 3 and 5, but also even beyond 5 years of age (see e.g., Jacqui A. Macdonald, Miriam H. Beauchamp, Judith A. Crigan & Peter J. Anderson (2014) Age-related differences in inhibitory control in the early school years, Child Neuropsychology: A Journal on Normal and Abnormal Development in Childhood and Adolescence, 20:5, 509-526).

Lines 71/72: I am not entirely convinced by the authors’ conclusion that “no clear pattern has emerged between the self-control abilities of tool-using and non-tool-using species”. While it is true that no conclusion can yet be drawn from studies looking into self control in non-tool-using contexts (because – as the authors rightly state – species have rarely been tested on the same tasks), the studies on animals’ performance in tasks involving delayed gratification in a *tool*-*use* context (lines 86-100), might be somewhat clearer. It seems to emerge that animals of different species are able to delay gratification and to select a tool over getting an immediate reward *regardless* of whether the animal belongs to a tool-using species or not. The authors should discuss the possibility that the existing literature might suggest that there is *no* relationship between self-control abilities and being a tool user (or not) in delay of gratification tasks involving tool use. Alternatively, if the authors think the results of the previous studies are truly inconclusive, they should explain more clearly why they think that (if there is a reason other than that different tasks were used).

A related point, what is missing before the paragraph starting in line 68 is a bit of theoretical background, i.e., whether researchers have had a priori expectations to find that tool-users have better self control (and if so, why they expect that).

Also related to this part in the text, I think it would be great to move the point stated in line 101 (that “very few studies have used comparable methodology”) further up, to line 71, i.e., the authors could say that no clear pattern has emerged yet, also because different methodology has been used. This is an important point and can be made way earlier in the text.

Lines 105/106: “found differences between human and non-human species’ performances, which suggest that the task may not measure the ability that it is designed to test”. This sentence is a bit too vague – could you please specify what kind of differences were found? After all, you seem to describe not mere performance differences, but actually differences in the how the task is approached/perceived by the subjects?
Relatedly, line 106, “may not measure the ability that it is designed to test”. While possible, this is not always true. Isn’t it more likely that when task differences occur (see the performance differences in the trap tube task which the authors mention) that the task is indeed measuring what it is supposed to measure in *one* species, but not the in the other one? It seems that in most of the cases, the issue is that a task has been designed to measure an ability in one species and then *fails to transfer* rather than it not measuring the desired ability in either of the species. Thus, I suggest to rephrase the sentence to reflect this possibility.

At some point in the introduction it might be nice to have a brief explanation of why we should know about decision-making abilities in a tool-use context specifically. While the authors describe that a tool-use context is special (by having more relational complexity and more work-effort), they miss to describe why it is interesting to investigate delay of gratification in a tool-use context. What insight can this give us on different species’ cognition? Is it because of the greater ecological validity? One could argue that if one is interested in self-control only, one should revert to non-tool-use tasks as these would not introduce another potential source of variance. I would like to see the authors countering this potential criticism.

Lines 125/126: More information should be given on the different onset times of delayed gratification and tool functionality - the link to what has previously been investigated is rather short. Some more explanation here might also help to justify the choice of the age range, which is missing in the methods section (see below).

Lines 126-128: Unlike for the non-human species, the authors state no directed hypotheses about children’s performance in the task. The authors should explain why not. Can one deduce from the previous literature on the different onset times of tool functionality understanding on the one hand and delay of gratification on the other hand help to formulate a hypothesis?

Methods

The methodology is sound. The statistical design seems mostly appropriate, however I have a few questions/comments on the design and analysis (see below). The procedures have mostly been described sufficiently, however, some details are missing that allow the study to be replicated (see below).

Line 110: Please provide a justification for why this age range of children (3-5) was chosen.

Line 135: how was the sample size for the crows determined? Did the authors have a target number?

Line 139: The authors state that they were testing juvenile and adult crows. What is the implication of this? Is it assumed that juveniles already have the same abilities as adults? Why did the authors not decide to only test adult crows? Does a mix of juveniles and adults not matter? How much were practical issues decisive? More information on these questions should be given in order to help the reader understand whether having juveniles and adults in the sample is not an issue at all or should be seen as a concern.

Line 142: can the authors add information on how big the caught family groups were?

Line 156: how was the sample size for the child sample determined? Was there an a-priori sample size calculation?

Line 160: how was the sample size for the adult sample determined?

Line 171: would the authors consider moving figure S1 to the main manuscript? It would benefit the understanding of the experiment tremendously to have a picture of the apparatuses readily available. If not, I would like to ask the authors to move S1 to the beginning of their supplementary materials, before the result tables (just to stay in chronological order).

Lines 173-181: the information that two different stick apparatuses were used for humans and NC crows should come *before* the description of the apparatuses; just makes this section easier to understand.

Line 179: again, it would be nice if figure S1 was in the main manuscript rather than in the supplementary material.

Line 179: as the authors refer to S1a Fig, they should also refer to S1**b** Fig (e.g., in line 175).

lines 335/336: please add the explanation for why subjects were divided into two subsets with a different order of conditions.

347: please explain why an unusually low number of videos (10%) were coded for reliability – usually 20 – 25% are used?

355-360: Why were no random slopes (for condition, apparatus, trial number) included into the model? According to Barr, Levy, Scheepers and Tily (2013) one should try to construct a maximal full model in order to keep type 1 error rate minimal. I suggest re-running the models and – if they lead to non-convergence – simplifying the models according to a pre-determined process.

References: Barr, D. J., Levy, R., Scheepers, C., & Tily, H. J. (2013). Random effects structure for confirmatory hypothesis testing: Keep it maximal. Journal of Memory and Language, 68(3), 225-278.

There is no report on model stability nor any model diagnostics checks. These analyses have to be run and included in the main manuscript or the supplementary material.

Line 358/359: There are two issues with age.

1. Usually in developmental studies on young children, when age is entered as continuous variable into models, this means that age in months is used. If children are assigned a year value (3, 4, 5) this is usually referred to as using age as a categorical variable. As I understand, in the current manuscript children were assigned a year value, which is equivalent to using a categorical variable. Why did the authors choose to label this a continuous variable? The variable with the three levels is arguable too coarse to label it continuous?
2. Why in the first place did the authors decide to bin age into the three levels? I suggest using the truly continuous variable (age in years and months) instead. Why binning one of the few continuous variables that developmental psychologists have and thus losing information? In addition, binning age implies that a child aged 3y11m is different from a child aged 4y1m, whereas a child aged 4y1m is put into the same category as a child with 4y11m. This seems unintuitive. It is suggested to rerun the analyses with age as a continuous variable.

Results

The results were partly not easy to understand. Some information in the text is overlapping with information given in table 1. It remained unclear why some results were presented in the main text and others were moved to the supplementary section. Table 2 is also rather difficult to grasp (see below).

Lines 430/431: relating to the comment above on the measurement level on age, it is unclear why the authors present the results on age for both age as a truly continuous (age in months) and a categorical (age in years) variable. The authors should consider using only the continuous variable from the start or to clearly justify their decision in the methods section.

Lines 431-432: Do the authors have any references in favour of excluding subjects in order to create more distinct age classes? To me, it seems that there is no reason to get rid of datapoints (especially given that the sample is not very large to begin with). In order to circumvent excluding datapoints, one could just go with age as a continuous variable for all analyses.

Line 363: it is unclear which variables in the GLMM are the control variables. I assume that one is gender, but it’s unclear whether for the children age was treated as a control variable or predictor. Please specify.

Line 364: the reference given after this sentence seems to be wrong or at least it is unclear how it relates. The reference “52” given below in the reference list is “Göckeritz S, Schmidt MF, Tomasello M. Young children's creation and transmission of social norms. Cognitive Development. 2014;30:81-95.” Please correct and check the correctness of the references throughout the text.

Lines 365-367: It is not explained what was analysed using the two-tailed statistics; which comparisons were made? Please clarify.

Line 367: Please explain why for non-parametric tests a different software was used.

Line 369: could the authors clarify which question the exact two-tailed Binomial tests address? “assessing success rates” is quite vague and could also be achieved by studying the descriptive data.

Lines 382-387 and following (e.g. lines 411-412)/Table 1: Would the authors consider adding to Table 1 the performance for each apparatus separately? In the text, the data for both “across apparatuses” and “for each apparatus separately” are presented together, and in between there are references to the table. It was a bit odd that the table would only present part of the data.

Lines 382-407 seem to describe in large parts what is displayed in table 1 and Figure 2. This is repetitive and the authors should consider presenting these results in a more concise manner, e.g., by cutting some parts of the text. In any case, I would recommend removing those sentences explaining which was the correct choice in which condition. This was already explained in the methods section and in Figure 1.

Line 426: since the interaction between age and condition is significant, the main effect of age is not interpretable (as it is dependent on condition) and should thus not be presented. Instead, only the interaction effect should be reported.

Line 428: “success rate increases with age” – this sentence implies a main effect of age, but this is not what was found, Please rephrase so that the text correctly describes the relationship between age and condition. For this, displaying the results visually would be beneficial for the understanding of the reader.

Line 430: Why was the interaction not further investigated? The methods section mentioned the posthoc tests using the multcomp package, but the results from these analyses don’t seem to appear in the results section. Please add.

Lines 433-434. “Across all conditions, only the 4 and 5-year old children selected correctly above chance across all trials”; later on the authors explain “3 to 5-year olds did not select correctly above chance in the tool functionality condition.” These two sentences are contradicting and the first sentence is potentially misleading. It suggests that 5-year-olds performed above chance in all conditions, while a look into the table and the second sentence exclude the tool functionality condition. To avoid a misunderstanding and to highlight the fact that the results are not as clear-cut as the first sentence might suggest, this first sentence should be modified accordingly to accurately describe the data.

Table S7: would the authors consider including the data displayed in this table into Table 2 in the main text? This way, the performance in all conditions could be more easily compared by the reader.

Table 2: Instead of displaying the uncorrected p-values and then adding “NS” if the values got insignificant after the Bonferroni correction, would the authors consider just presenting the p-values obtained after the correction? This would make it a lot easier to quickly grasp the results pattern when looking at the table. What benefit does including the non-corrected values have?

Lines 467-477 and Table 3: Why is children’s performance in the tool functionality condition displayed collapsed across the two subconditions? It was found that children indeed chose correctly when the tool was functional, so the conclusion that children performed poorly in the tool functionality condition might not accurately describe their ability to perceive affordances and use tools.

Discussion and conclusions

Line 497: it is stated that the study showed that the studied species were able to make profitable decisions in tasks requiring delayed gratification in a tool-use context. This phrasing suggests that all studied conditions involved a delay of gratification component, which does not seem to be the case. The authors should carefully rephrase this sentence at the beginning of this study; this is an important sentence which many readers will read first after the abstract. As it is formulated at the moment, the sentence suggests that the study was a delay of gratification /self-control study, which I wouldn’t have called it. The subsequent sentence indeed states that the studied populations “were able to flexibly select between reward items of differing quality and tools […] relative to the context of each condition.” – this has nothing to do with delay of gratification and shows that the focus of the paper was broader (effective decision-making). This is also evident by how the following sections in the discussion are structured: First the results on tool functionality are presented, then on motivation, and only then the delay of gratification part is presented. The authors need to be clearer in describing whether self-control/delay of gratification is their main focus of the paper or just one of several (and if there are several aspects, are the other ones related to the requirements of effective decision-making or as these aspects on the motivations and perceptions of affordances in tool-use contexts?). This is unclear in both the discussion and the introduction and needs to be more streamlined.

Linea 514/515: the authors state that children failed in the tool functionality condition. However, if I understood the results section correctly, the results could be differentiated between the two sub-conditions (i.e., when the tool was functional vs not functional). This is an interesting finding and should be acknowledged in the discussion. The way this is currently formulated does not represent the data accurately (very broad brush).

Lines 522-527: Can the authors elaborate on why the “birds’ performance at the four tasks that the 3-year olds struggled with, and particularly the motivation condition, which 4- 5-year olds and orangutans also struggled with” suggest that the cognitive demands of these conditions were “non-trivial”? Could these results also just be explained by a strong bias of choosing the tool in the children, which would not comment on whether or not the cognitive demands are difficult (i.e., it is about motivation, not cognition)?

Lines 533-534: “4-5-year olds, though not 3-year olds, appear able to overcome the desire to immediately select the tool”. This might suggest that 3-year-olds lack the ability to overcome this desire. However, the authors should consider that the reason why the 3-year-olds struggled could eb that they perceived the task differently, for example as a task in which they try to explore the novel materials and tools instead of one in which getting the reward was the primary goal.

Line 555: another reason why children performed badly at the tool functionality task could not only be the “drive to give it a go”, trying to make the non-functional tool work, but it could also be that the children know well in advance that the tool will not work, but select it because it is fun manipulating the tool and exploring the apparatus with it. Could the authors provide information on what the children were attempting to do with the tool (did they just select it, were they trying to actually get the reward?) or were children not given the chance to try out the non-functional tool in the test?

In the result section it was found that performance sometimes differed between the two apparatuses. This should be at least mentioned in the discussion, preferably also discussed.

The authors provide possible explanations of why there yould be a difference between crows and cockatoo performance, but an explanation of what the difference between in the conditions in which the 3-year-olds struggle and the birds succeed seems not to be given? The authors should also comment on this difference.

The authors have presented many different potential explanations for the findings in the various conditions. However, given the emphasis of self-control in the introduction, could the authors make a concluding statement in the discussion on what was learned with regard to the self-control abilities of the studied groups, if anything at all can be said with certainty?

It would be nice if the authors could make a bit clearer in the discussion that xyz was their main focus and abc is what they found. At the moment, the discussion is rather driven by what has been found, e.g., in lines 574 and following the results from the tool selection quality allocation condition are presented as the most prominent finding and for the first time the ability to process information is discussed, which doesn’t seem to be strong focus of the paper at all.

Minor issues:

- Line 50: comma before „and the quality”
- Line 51: I suggest to start a new paragraph for the sentence beginning with “One aspect that underlies”; that way, the first paragraph describes decision-making, whereas the second one focuses on self-control/delay of gratification, and will help the reader follow the structure and arguments of the paper.
- Line 58: another good review article for the development of inhibitory control in children, which the authors could refer to, is: Garon, N., Bryson, S. E., & Smith, I. M. (2008). Executive Function in Preschoolers: A Review Using an Integrative Framework. *Psychological Bulletin*, *134*(1), 31–60.
- Lines 64-67: The word “therefore” seems to be misplaced. “Therefore” seems to imply that the argument that decision-making involving tool use may require increased levels of complexity results from what has been described in the previous sentence; however, this is not the case. Rather, there seem to be two separate issues involved in decision-making involving tool use: 1) tool use requires delay of gratification and 2) tool use requires an increased level of relational complexity, as well as work-effort. Relatedly, the next sentence starting with “For example” doesn’t seem to be an example of what has been explained in the previous sentence. How is the need to pay attention to tool functionality linked to an increased relational complexity? I understand that paying attention to the tool per se (compared to only paying attention to the reward) increases relational complexity, but this seems to be regardless of whether or not attention is paid to tool functionality. Neither does the given example fit to the statement that tool use may require additional work-effort. I suggest that the authors rephrase this or make it clearer in how far their example represents either of the two issues mentioned in the previous sentence (increased relational complexity or additional work-effort).
- Lines 68/69: “has been found in various non-human species, including primates and birds” – the nomenclature is not quite correct here – the authors use the label “species” and when they continue with their examples (birds, primates) the phrasing seems to suggest that primates and birds are labels for species. Please try to be as scientifically correct as possible and try to rephrase.
- Lines 77 and 79: “tool-making” – this is the first time the authors use the label “tool-making” – they started the paragraph differentiating between tool-*using* and non-tool-*using* animals and then there is the word “tool-making”. It doesn’t become clear whether these labels are used interchangeably (they shouldn’t). Is there a reason for the switch in the labels? If so, this has to be explained, as otherwise this leaves the reader potentially confused. If there is no reason, I suggest using just one label throughout the manuscript (presumable “(non-) tool-*using”* as the paper focuses on tool use, not on tool manufacture) in order to not introduce any ambiguity or confusion.
- Line 84: please insert “;” after “i.e.”
- Line 110: comma after “humans”?
- Line 144: comma after “branches”?
- Line 122: “require” instead of “requires”?
- Line 170: remove comma in “box, was”
- Line 170: comma after “i.e.”
- Line 214: “Supplemental Video” – please specify which video (2 in this case)
- Lines 351/352: please provide references for the R packages
- Line 367: “data were” instead of “data was”
- Line 372: “i**n** S1 Table” instead of “i S1 Table”
- Lines 377/378 and 425-427: replace “X” with “χ”
- Line 378 and 426-427: italicize “p”-values
- Line 378: insert space between “<” and “0.001”
- Lines 378/379 and 426-427: remove the first zero in “0.001” – as the value is bound between 0 and 1, there does not need to be a 0 before the decimal point
- Table 1: remove zeros before the decimal points
- Table 1: It is not explained what “S1&2” stands for
- Lines 387-392: “For the motivation condition, the correct choice was the immediately available most preferred reward over the functional tool, with the same most preferred reward inside the apparatus. For the quality allocation condition, the correct choice was the immediately available most preferred reward over the tool when the least preferred reward was inside the apparatus, or the tool over the least preferred reward, when the most preferred reward was inside the apparatus.” This was already explained in the methods section and it’s unclear why this is repeated here. Delete.
- Table 2:
  - please remove the zero before the decimal point all in p-values.
  - Insert a space between “<” and the respective p-values
  - The authors write “<.0001” several times. The convention is to use .001 (3 decimal places and the authors should consider changing this.
  - In the column “tool functionality”, “P” should be “p”
  - Why are some p-values italicized? This is not explained in the figure caption and needs to be added.
- Line 451: “-“ after “3” and “4”
- Line 457: “-“ after “3”
- Lines 463-464: This heading is too vague. Isn’t this section more about a comparison? The performance of the crows and the humans was already presented in the previous sections, so labelling this section “performance in NC crows, …children, and adult humans” is potentially confusing. I suggest rephrasing the heading so that it is more specific and describes exactly what the following section is about
- Lines 456-466: “we illustrate the performance…with that” – this seems grammatically incorrect. Better: we compare performance…with that?
- Line 497: remove comma after “humans”
- Line 519: “succeed” instead of “success”
- Line 526: “species” instead of “species’”
- Line 539: comma after “mentality”
- Line 584: “three” instead of “3”
- Line 684: the “S” in “Psychological science” should be capital letter
- Supplementary Material S3 and S4 Table: In the caption of both tables, replace “models” with “model”
